# Supplementary material for: Content-rich biological network constructed by mining PubMed abstracts
Source: BMC Bioinformatics. 2004 Oct 8;5:147. doi: 10.1186/1471-2105-5-147 (PMC528731; doi:10.1186/1471-2105-5-147)
Supplement: Additional File 2 — The original results of the above study (non-essential files are deleted to keep the file size under the limit set by BMC bioinformatics). [file 1471-2105-5-147-S2.bz2 › chilibotAdditionalFile2/dip30/700ID10869074E18033/html/MIND_MINC.html]

 


 **MIND** and **MINC** 
  
Found 51 abstracts in PubMed, retrieved 30.  
 

 What does Google say? 
 PDF only 
| .edu only 

---

**Interactive relationship** (e.g. stimulation, inhibition, etc)

**Inhibitory relationship**- **MinC**  mediated division inhibition can also be activated in a  **MinD**  independent fashion by the DicB protein of cryptic prophage Kim.  Ref: 12003935 J Bacteriol, 2002
- These studies suggest that  **MinD**  Ng is involved in inhibiting gonococcal cell division, likely in conjunction with  **MinC**  Ng.  Ref: 11591668 J Bacteriol, 2001
- Several mutations affecting the mid to C terminal parts of  **MinD**  led to a protein probably unable to interact with DivIVA, but that could still stimulate division inhibition by  **MinC** .  Ref: 11886553 Mol Microbiol, 2001
- In Escherichia coli, site selection is dependent on  **MinC** ,  **MinD**  and MINE  **MinC**  acts, with  **MinD** , to inhibit division at sites other than the midcell by directly interacting with FTSZ Here we report the crystal structure to 2.2 A of  **MinC**  from Thermotoga maritima.  Ref: 11350934 EMBO J, 2001
**Neutral relationship**- **MinD**  and MinE regulate  **MinC**  activity by modulating its cellular location in a unique fashion.  Ref: 11285221 EMBO J, 2001
- iii both  **MinC**  and MinE can be recruited to  **MinD** .ATP decorated vesicles.  Ref: 12533449 J Bacteriol, 2003
- Here, we present evidence that the binding of D  **MinC**  to  **MinD**  endows the  **MinC**   **MinD**  complex with a more specific affinity for a septal ring associated target in vivo.  Ref: 12003935 J Bacteriol, 2002
- Also, it is argued that binding of  **MinD**  to the membrane activates  **MinC** .  Ref: 12486056 J Bacteriol, 2003
- Analysis of  **MinC**  reveals two independent domains involved in interaction with  **MinD**  and FtsZ.  Ref: 10869074 J Bacteriol, 2000
- **MinC**  oscillates along with  **MinD**  such that the time integrated concentration of Z  **MinC**  at the membrane is minimal, and hence the stability of FtsZ polymers is maximal, at the cell center.  Ref: 12003935 J Bacteriol, 2002
- We observed that  **MinD**  dimerized in the presence of ATP and interacted with  **MinC** .  Ref: 12486056 J Bacteriol, 2003
- In the presence of a phospholipid bilayer,  **MinD**  bound to the bilayer and recruited  **MinC**  in an ATP dependent manner.  Ref: 12486056 J Bacteriol, 2003
- A conserved sequence at the C terminus of  **MinD**  is required for binding to the membrane and targeting  **MinC**  to the septum.  Ref: 12519187 Mol Microbiol, 2003
- These results suggest that MinE induces a conformational change in  **MinD**  bound to the bilayer that results in the release of  **MinC** .  Ref: 12486056 J Bacteriol, 2003
- In contrast,  **MinC**  was unable to displace MinE bound to the  **MinD**  bilayer complex.  Ref: 12486056 J Bacteriol, 2003
- ... the C terminal domain D  **MinC**  is required for dimerization as well as for interaction with the  **MinD**  protein.  Ref: 12003935 J Bacteriol, 2002
- Targeting of D  **MinC**   **MinD**  and D  **MinC**  DicB complexes to septal rings in Escherichia coli suggests a multistep mechanism for  **MinC**  mediated destruction of nascent FtsZ rings.  Ref: 12003935 J Bacteriol, 2002
- **MinD**  plays a central role in this process, as it positions and activates  **MinC**  and is dependent on DivIVA for its own positioning at the poles.  Ref: 11886553 Mol Microbiol, 2001
- **MinC**  dynamics involves a membrane association dissociation cycle that is driven by the activities of the  **MinD**  ATPase and the MinE topological specificity factor, which themselves undergo coupled oscillatory localization cycles.  Ref: 12533449 J Bacteriol, 2003
- Thus,  **MinD**  does not merely attract  **MinC**  to the membrane but also aids  **MinC**  in specifically binding to, or in close proximity to, the substrate of its Z  **MinC**  domain.  Ref: 12003935 J Bacteriol, 2002
- By using computer simulations we show that Min protein dynamics can be described accurately by using the following assumptions i the  **MinD**  ATPase self assembles on the membrane and recruits both  **MinC** , an inhibitor of Z ring formation, and MinE, a protein required for  **MinC**   **MinD**  oscillation, ii a local accumulation of MinE is generated by a pattern formation reaction that is based on local self enhancement and a long range antagonistic effect, and iii it displaces  **MinD**  from the membrane causing its own local destabilization and shift toward higher  **MinD**  concentrations.  Ref: 11734639 Proc Natl Acad Sci U S A, 2001
- We show that cytoplasmic  **MinC**  is poised near the monomer dimer equilibrium and propose that it only becomes entirely dimeric once recruited to the membrane by  **MinD** .  Ref: 11673440 J Bacteriol, 2001
- In the presence of ATP  **MinD**  binds  **MinC**  and goes to the membrane the ATPase is not stimulated and the complex is stable.  Ref: 12675792 Mol Microbiol, 2003

**Non-interactive relationship** (e.g. studied together, co-existance, homology, etc.)

- BACKGROUND In Escherichia coli, the cell division site is determined by the cooperative activity of min operon products  **MinC** ,  **MinD** , and MinE.  Ref: 11566131 Structure Camb , 2001
- The joint overexpression of  **MinC**  Ng and  **MinD**  Ng from a shuttle vector resulted in a significant enlargement of gonococcal cells, while cells transformed with plasmids encoding either  **MinC**  Ng or  **MinD**  Ng alone did not display noticeable morphological changes.  Ref: 11591668 J Bacteriol, 2001
- **MinC**  is cytoplasmic and fails to block FtsZ assembly in the absence of  **MinD** , indicating that recruitment of  **MinC**  by  **MinD**  to the membrane enhances Z  **MinC**  function.  Ref: 12003935 J Bacteriol, 2002
- The results suggest a multistep mechanism for the activation of  **MinC**  mediated division inhibition by either  **MinD**  or DicB and further expand the number of properties that can be ascribed to the Min proteins.  Ref: 12003935 J Bacteriol, 2002
- The results also indicate that MinE stimulated dissociation of  **MinC**  from the  **MinC**   **MinD** .ATP membrane complex can, and may, occur prior to hydrolysis of the nucleotide.  Ref: 12533449 J Bacteriol, 2003
- and v MinE stimulates dissociation of  **MinC**  from  **MinD** .ATP membrane complexes, even when ATP hydrolysis is blocked.  Ref: 12533449 J Bacteriol, 2003
- **MinD**  recruits  **MinC**  to the membrane, and MinE induces  **MinC**   **MinD**  to oscillate rapidly between the membrane of opposite cell halves.  Ref: 11285221 EMBO J, 2001
- To clarify the functional role of the bacterial cell division inhibitor  **MinD**   [ **MIND** ] , which is a membrane associated ATPase that works as an activator of  **MinC** , we determined the crystal structure of a Pyrococcus furiosus  **MinD**  homologue complexed with a substrate analogue, AMPPCP, and with the product ADP at resolutions of 2.7 and 2.0 A, respectively.  Ref: 11296216 EMBO J, 2001
- With  **MinC** ,  **MinD**  forms a non specific inhibitor of division, that interacts with FtsZ.  Ref: 11248256 FEBS Lett, 2001
- Introduction of a negatively charged amino acid decreased membrane binding of  **MinD**  and its ability to activate  **MinC** .  Ref: 12867440 J Bacteriol, 2003
- In Escherichia coli, selection of the correct mid cell site is mediated by the  **MinC** ,  **MinD**  and MinE proteins.  Ref: 11296216 EMBO J, 2001
- This finding correlates well with the previously observed oscillatory movement of  **MinC**  and  **MinD**  in short and long cells.  Ref: 10931285 Mol Microbiol, 2000
- Addition of MinE to the MinCD bilayer complex resulted in release of both  **MinC**  and  **MinD** .  Ref: 12486056 J Bacteriol, 2003
- Recruitment of  **MinC** , an inhibitor of Z ring formation, to the membrane in Escherichia coli role of  **MinD**  and MinE.  Ref: 12486056 J Bacteriol, 2003
- DicB shows little homology to  **MinD** , and how it stimulates  **MinC**  function has been unclear.  Ref: 12003935 J Bacteriol, 2002
- The activity of the promoter regions of the cell division genes ftsZ, ftsE,  **minC** ,  **minD**  and minE from Neisseria gonorrhoeae Ng was studied under different environmental conditions using lacZ translational fusions.  Ref: 11763238 Res Microbiol, 2001
- This positioning is achieved through a rapid oscillation of  **MinC**  from pole to pole, a process requiring  **MinD**  and MinE.  Ref: 11430835 Mol Cell, 2001
- In rod shaped bacteria such as Bacillus subtilis, division site selection is mediated by  **MinC**  and  **MinD** , which together function as a division inhibitor.  Ref: 11886553 Mol Microbiol, 2001
- Recent studies have shown that MinE, previously thought to form a static ring near the division site at the midcell position, actually joins  **MinC**  and  **MinD**  in their rapid oscillation between the cell poles.  Ref: 11378404 Curr Biol, 2001
- The Min system comprises  **MinD**  and  **MinC** , which form an inhibitor complex and, in Bacillus subtilis, DivIVA, which ensures that division is inhibited only in the polar regions.  Ref: 12492852 Mol Microbiol, 2003
- These results demonstrate that  **MinC**  consists of two independently functioning domains an N terminal domain capable of inhibiting FtsZ assembly and a C terminal domain responsible for localization of  **MinC**  through interaction with  **MinD** .  Ref: 10869074 J Bacteriol, 2000
- Correct positioning of the division septum in Escherichia coli depends on the coordinated action of the  **MinC** ,  **MinD**  and MinE proteins.  Ref: 11062554 Nat Struct Biol, 2000
- Positioning of the midcell division plane within the bacterium E. coli is controlled by the min system of proteins  **MinC** ,  **MinD** , and MinE.  Ref: 11800919 Phys Rev Lett, 2001
- In Escherichia coli, this process depends crucially on the proteins  **MinC** ,  **MinD** , and MinE.  Ref: 11806906 Biophys J, 2002
- MinE assembles into a membrane associated ring structure near midcell and directs the localization of  **MinD**  and  **MinC**  into a membrane associated polar zone that undergoes a characteristic pole to pole oscillation cycle.  Ref: 12093736 EMBO J, 2002
- Accurate positioning of the division septum at the equator of Escherichia coli cells requires a rapid oscillation of  **MinD**  ATPase between the polar halves of the cell membrane, together with the division inhibitor  **MinC** , under MinE control.  Ref: 12482939 Proc Natl Acad Sci U S A, 2002
- Cell division inhibitors SulA and  **MinC**   **MinD**  block septum formation at different steps in the assembly of the Escherichia coli division machinery.  Ref: 10931335 Mol Microbiol, 2000
- **MinD**  localizes to the cytoplasmic face of the inner membrane in Escherichia coli, and its association with the inner membrane is a prerequisite for membrane recruitment of the septation inhibitor  **MinC** .  Ref: 12424340 Proc Natl Acad Sci U S A, 2002
- The release of  **MinC**  did not require ATP hydrolysis, indicating that MinE could displace  **MinC**  from the  **MinD**  bilayer complex.  Ref: 12486056 J Bacteriol, 2003
- Similar to the results obtained with  **MinD** , we find that DicB interacts directly with D  **MinC** , that the D  **MinC**  DicB complex has a high affinity for some septal ring target s,.  Ref: 12003935 J Bacteriol, 2002
- The Min system of Escherichia coli directs cell division to the mid cell by a mechanism that involves the dynamic localization of all of its three constituent proteins,  **MinC** ,  **MinD**  and MinE.  Ref: 12368455 Microbiology, 2002
